# Acromegalic gigantism, physicians and body snatching. Past or present?

**DOI:** 10.1007/s11102-012-0389-5

**Published:** 2012-04-06

**Authors:** Wouter W. de Herder

**Affiliations:** Department of Internal Medicine, Sector of Endocrinology, Erasmus MC, ‘s Gravendijkwal 230, 3015 CE Rotterdam, The Netherlands

**Keywords:** Acromegaly, Gigantism, Famous persons

## Abstract

The skeletons of 2 famous acromegalic giants: Charles Byrne (1761–1783) and Henri Cot = Joseph Dusorc (1883–1912) and the embalmed body of the famous acromegalic giant Édouard Beaupré (1881–1904) all ended up in the medical collections of museums despite the fact that these patients had never donated or even refused to donate their corpses, nor had their relatives given permission. The corpse of the acromegalic giant John Aasen (1890–1938) was voluntarily donated to a physician annex collector of trivia from acromegalic giants. The autopsy on the acromegalic giant John Turner (1874–1911) was performed during his funeral ceremony without the relatives being informed. Only recently, the acromegalic giant Alexander Sizonenko (1959–2012) was made a financial offer during his life in exchange for his body after his death. The case-histories of these 6 patients and also the circumstances that led to the (in-) voluntary donation of their bodies are reviewed.

## Introduction

In a recent article in the British Medical Journal by Len Doyal, emeritus professor of medical ethics, and Thomas Muinzer, lawyer, the authors make the case for the removal of the skeleton of Charles Byrne from the Hunterian Museum in the Royal College of Surgeons in London, U.K., where it is on display. Subsequently it should be buried at sea, according to Charles Byrne’s original wish [1]. The article has raised a very lively discussion [2–4].

## Case histories

**Fig. 1:**
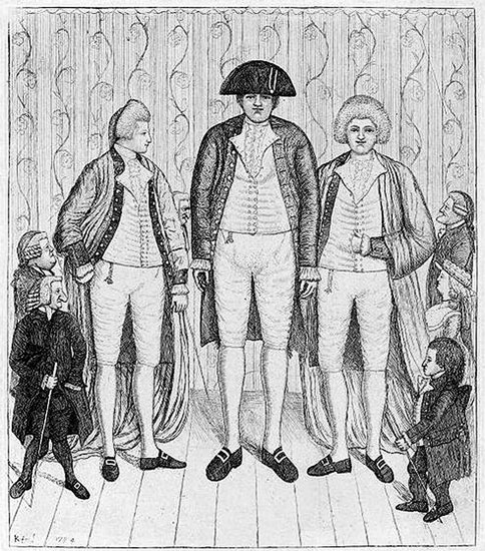
Charles Byrne (1761–1783) and the “giant” Knipe brothers, together with Andrew Bell, Baillie Kid, James Burnett – Lord Monboddo and William Richardson. Etching & aquatint by John Kay, 1784. Original in National Portrait Gallery, London, UK. Picture from the collection of Wouter W. de Herder

(1) Charles Byrne (Fig. 1) was born in 1761 in Littlebridge (Northern) Ireland. During childhood he “grew like a cornstalk”. He reached a final height of 2.31 m. (7 ft. 7 in.) tall. As a teenager, Charles Byrne was exhibited at local fairs and markets. His manager was convinced that Charles Byrne, alias the “Irish Giant”, could attract a much larger audience and thus make more money in England. After 1782, Charles Byrne had adopted the name “O’Brien” and started entertaining the public in London.

Rumours of Byrne’s financial success also inspired other giants to try their luck in London. The “Gigantic Twin Brothers” Knipe were from a village in Ireland just a few miles from Charles Byrne’s birthplace. They once even claimed to be cousins of Charles Byrne. Surprisingly, this might have been true as well (see below). Another Irish giant, Patrick Cotter, was born 1760 in Kinsale, County Cork, Ireland. He also chose “O’Brien” as his stage name and was also exhibited at side shows in nearby towns. He was known as the “Bristol Giant” and the “Irish Giant”. He died at Hotwells, Bristol on 8 September 1806 [5]. In December 1972, his bones were retrieved from his grave and it was determined that, while alive, he measured approximately 2.46 m. (8 ft. 1 in.) [5].

Around 1783, the public became fed-up with Charles Byrne–O’Brien and began to frequent other entertainments. Overtaken by his fame and wealth, Charles Byrne had started drinking huge quantities of alcohol. He possessed 2 banknotes, one £700 note and a £70 note. Charles Byrne foolishly chose to carry these banknotes on him. According to newspaper reports he was out drinking when he was robbed of his £700 banknote. In less than 1 year after his arrival in London, Charles Byrne had lost almost everything. It is also known that at this point he had contracted “consumption” (tuberculosis). The combination of his alcoholism and tuberculosis weakened him. As a result, by May 1783, Charles Byrne realized that he was a dying man.

Death itself was not Charles Byrne’s greatest fear but rather it was the physicians. A number of them were eager to obtain his body after his death for dissection. One of them was the surgeon Dr. John Hunter (1728–1793) [6], who is presently also known as the “Godfather of Modern Surgery” [7–9].

Determined to stay out of the physicians’ hands and especially those of Dr. John Hunter, Charles Byrne started making precautions. After his death, his body was to be sealed in a lead coffin and to be watched day and night by his loyal friends until it could be sunk deep in the sea. Using the remains of his saving money, Charles Byrne prepaid the undertaker to ensure that his will would be carried out. Meanwhile, Dr. John Hunter had employed a detective to keep a close watch on Byrne’s whereabouts as well as on his deteriorating condition.

On 1 June 1783, Charles Byrne died at the age of 22 [10]. After obtaining an oversized coffin, Charles Byrne’s friends kept watch over the corpse for 4 days. First, they exhibited the enormous casket for money to the public. Finally, on 6 June 1783 they began their 75 miles voyage to transport the coffin with the corpse of Charles Byrne to the seaside town of Margate, England. Once they arrived, a boat was chartered and the massive coffin was plunged into the sea. Soon after, rumours speculating on what actually happened to Charles Byrne’s body began to circulate.

The precise details on how the remains of Charles Byrne came into Dr. John Hunter’s possession are not known [11]. The most popular account of what had happened is as follows: after receiving the news that Charles Byrne had died, Dr. John Hunter located the undertaker who was responsible for executing the will of Charles Byrne. Dr. John Hunter bribed the unscrupulous undertaker at the price of £500 to obtain the corpse of Charles Byrne. The undertaker’s accomplices switched the corpse of Byrne for stones in a barn outside a tavern on the route to the coast, while the undertakers were having a drink. The corpse of Charles Byrne was taken back to London and delivered to Dr. John Hunter. Dr. John Hunter was terrified of retribution from Charles Byrne’s friends. Wanting to escape from public scrutiny, the skeleton of Charles Byrne was put on display in Dr. John Hunter’s museum 4 years after his death, after the public’s interest in him had waned. Charles Byrne’s skeleton now still resides in the “Hunterian Museum” at the Royal College of Surgeons in London, UK.

However, Dr. John Hunter could also have been the first to describe pituitary enlargement in gigantism/acromegaly, if only he would have opened the skull of the Charles Byrne [12]. But, as stated by the famous neurosurgeon Dr. Harvey Williams Cushing (1869–1939)—the “Godfather of Neurosurgery”—, “his passion as a collector exceeded his thirst for knowledge” [13, 14].

In 1909, Dr. Harvey Cushing together with Sir Arthur Keith, the curator of the John Hunter museum in London (UK), opened the skull of Charles Byrne and demonstrated that the sella turcica was enlarged [10, 13–17].

Although Charles Byrne was 22 years old when he died, radiographic images of his wrist bones showed that the distal epiphysial lines were still open. In 1980, Drs. Alex(ander) M. Landolt and Milo Zachmann estimated his “bone age” at the time of his death to be only about 17, indicating that he was still growing at the time of death and this is suggestive that Charles Byrne was suffering from (hypogonadotropic) hypogonadism [18].

In 2010, Dr. Harvinder S. Chahal and co-workers from the Department of Endocrinology, Barts and the London School of Medicine, Queen Mary University of London, London UK extracted DNA from a tooth of Charles Byrne and identified a germ-line mutation in the aryl hydrocarbon–interacting protein gene (AIP). Four contemporary Northern Irish families who presented with gigantism, acromegaly, or prolactinoma have the same mutation and haplotype associated with the mutated gene. Using coalescent theory, they infer that these persons share a common ancestor who lived about 57–66 generations earlier [19].

**Fig. 2:**
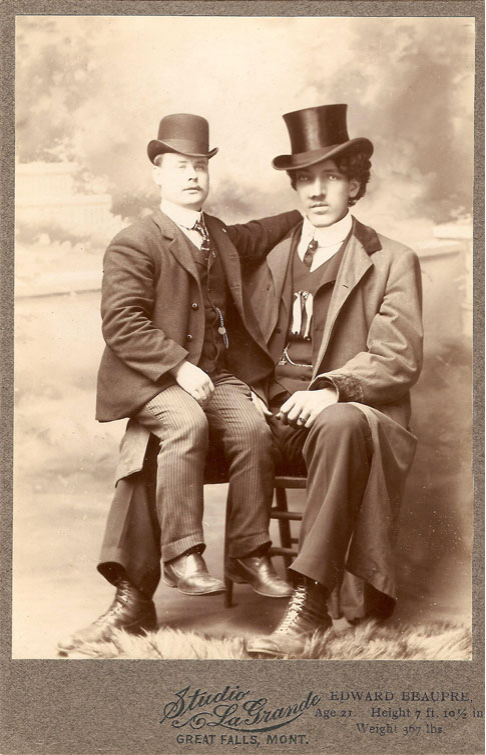
Édouard Beaupré (1881–1904) and a normal-sized man. Picture kindly provided by Warren Moulton

(2) Canada’s tallest man (Joseph) Édouard Beaupré (Fig. 2), was born on 9 January 1881 in Willow Bunch (Saskatchewan, Canada). He was the first-born in a very poor family which was to reach a total number of 20 children, of whom several died as infants. His parents, Gaspard Beaupré and Florestine Piché were of average height. His growth spurt started already after the age of 3. At the age of 9, he was already 1.83 m. (6 ft.) tall, at the age of 12 his height was 1.98 m. (6 ft. 6 in.) and at the age of 17 he had reached 2.16 m. (7 ft. 1 in.). His final height was 2.52 m. (8 ft. 3 in.) and he weighed 166 kg. (365 lbs.). Being unusually strong he became a much sought after cowboy and was herding cattle all across Canada. From the age of 17 (1897), Édouard Beaupré toured as “The Willow Bunch Giant” throughout Canada and the U.S.A. and worked in side shows, circuses and at fairs. His most heroic act at that time was to lift a horse weighing 363 kg. (800 lbs.) to shoulder height by crouching under it and extending its legs. In 1901 he started working for the manager Aimé Bénard who forced him to lift a weight of even 408 kg. (900 lbs.) causing severe damage to Édouard Beaupré’s joints and back. Eventually it was also Aimé Bénard who deceived him, turned him into a drunk, ruined his career and finally arranged for the disgusting exposure of Édouard Beaupré’s embalmed corpse after his death.

On 3 July 1904 he died while he was performing at the “World’s Fair” of St Louis, Missouri, U.S.A. The cause of death was pulmonary bleeding as a result of tuberculosis. A post-mortem examination was performed by Dr. Rutherford (Berchard Hayes) Gradwohl in St. Louis. His recorded length at that time was 2.44 m. (8 ft. 3 in.) and his weight was 170 kg (375 lbs.). His poor family could not, and his vicious manager Aimé Bénard would not pay for the body’s transportation or the funeral. The body was subsequently embalmed and displayed for money in the front window of “Eberle and Keyes Funeral Parlor” in St. Louis. Later the body was also displayed in the “Eden Museum” in Montreal, Canada, and from 1907 onwards the body was in the collection of the department of anatomy in the University of Montreal. A picture can be found in the publication of Dr. J.-Maurice Blais from 1967 [20, 21]. In this publication a skull radiograph shows an enlarged sella turcica [measuring 1.6 × 1.2 cm. (0.6 × 0.5 in.)] and overgrowth of the frontal bones [20, 21]. After a legal procedure, started by his relatives, the body of Edouard Beaupré was finally returned to his family in 1989 and subsequently cremated on September 28, 1989 in order to prevent future exposition. On July 7, 1990, his ashes were buried under the lawn near his statue in front of the Willow Bunch Museum [22].

**Fig. 3:**
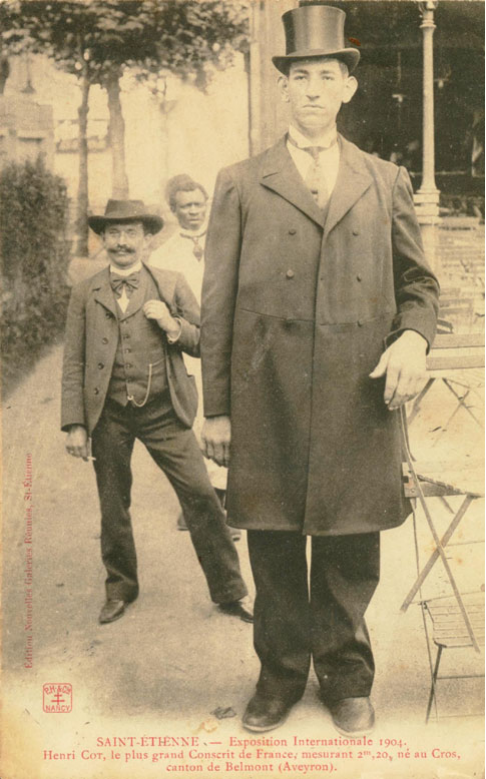
Henri Cot = Joseph Dusorc (1883–1912). Picture from the collection of Wouter W. de Herder

(3) Henri Joseph Cot (Fig. 3) was born at the village Le Cros, close to Belmont sur Rance in the Aveyron district, France, on 30 January 1883 [23]. He was the 7th and youngest child in a very poor farmer’s family. All other family members were of average size. Initially, his physical development was normal. His growth spurt started before the age of 8 years. At the age of 8 years, he was already 1.50 m. (4 ft. 11 in.) tall, at the age of 12 he was 1.70 m. (5 ft. 7 in.) tall and at the age of 16 years he was 1.95 m. (6 ft. 4¾in.) tall. At the age of 20 (in 1903), when he was called up for the army, his official height was 2.30 m. (7 ft. 6½ in.). However, he was not drafted because of this extreme height. He was named “le plus grand conscrit de France” [the tallest recruit in France] [23].

Because of his height and strength he was a celebrity in Le Cros and famous for showing his great strength. For example, he would lift a donkey onto his shoulders and carry it around the village. The stories about him attracted his future manager who eventually persuaded him to join the show business. He appeared at many fairs and shows, starting in France and Algeria. In 1906 he started touring the UK and Canada [23].

In order to launch a new career in show business in 1910, his manager rearranged his nickname, “le géant du Cros” [“the giant of Cros”] into a kind of anagram “dusorc”. Joseph Dusorc, as he now was called, grew a beard and was a so-called “veteran of the 3rd Napoleonic war” and was dressed-up as a drum master [23]. Joseph Dusorc was supposed to have been born in Garic, Béarn, France. His reported height varied from 2.38 to 2.48 m. (7 ft. 10 in.—8 ft. 4 in.) and his weight was 181 kg. (400 lbs.). For obvious reasons, his birth date was not given.

On 11 September 1912, Henri Cot/Joseph Dusorc died at the age of 29 years in Lyon, France. According to the newspaper “le Lyon Républicain” he died from a “cardiac embolism”. At his funeral in St. Affrique, France, several witnesses watched the coffin, weighing around 500!!?? kg. (1102 lbs.), being lowered down and immediately noticed that the coffin was shorter that the semi-official giant’s length. When the coffin tipped slightly a thud of stones could be heard. This convinced almost everybody who attended the funeral that the body of Henri Cot was not inside the coffin, but only stones [23]. Apparently after his manager had realized he had lost his income, he had the idea of selling Henri Cot’s body to a professor of medicine in Montpellier (France) to make money from him one last time. For this reason, the skeleton of Henri Cot/Joseph Dusorc is now exhibited in the medical school of Montpellier and labelled as “unknown giant”.

Dr. Charles D. Humberd (1897–1960) from Barnard, Mo, U.S.A. was a coroner of Nodaway County in northwest Missouri. He had a more than professional interest in giants, making him both famous and infamous in this field. Dr Charles Humberd owned hats, shoes, rings and other souvenirs of most of the circus and side show giants that toured the U.S.A. He became most infamous for his paper on the tallest giant ever, 2.72 m. (8 ft. 11.1 in.) tall Robert Pershing Wadlow (22 February 1918–15 July 1940) from Alton, Illinois, U.S.A., in the Journal of the American Medical Association (JAMA) and the $100,000 libel suit against him in the court of St Joseph, Mo, USA, over this article filed by Robert Wadlow and his family [24].

**Fig. 4:**
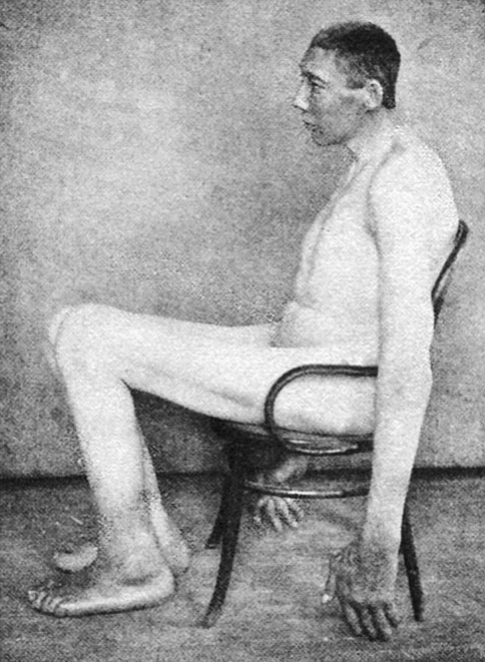
John Turner (1874–1911). Picture from the collection of Wouter W. de Herder

(4) In 1936, Dr. Charles D. Humberd published a paper discussing the true length of the acromegalic giant John Turner (231.1 cm, 7 ft. 7 in.—1874–1911) and stating that Dr. Harvey Cushing in his monograph “The Pituitary Body and its Disorders” (1912) [14] had made an 1 ft. (30 cm.) mistake in measuring the length of John Turner. Dr. Humberd noted that the height of Turner, case XXXII (surgical No. 25947—pp. 162–170) in Harvey Cushing’s monograph was recorded as 2.515 m. (8 ft. 3 in.), but it should have been noted as 2.21 m. (7 ft. 3 in.) [25] (Fig. 4).

Interestingly, the published post-mortem exam findings in 36-year-old John Turner are prefaced by a cryptic statement by Dr. Harvey Cushing, describing “inauspicious circumstances”. Namely, after Dr. Harvey Cushing had paid $50 to the undertaker, the autopsy was performed in Washington by his close colleagues, the surgeons Drs. Samuel J. Crowe and William Sharpe. But this was done without permission of the family in the undertakers establishment, while the funeral service was going on [14, 26, 27]!!!! According to Dr. Sam Crowe, this story shows how Dr. Harvey Cushing “would stop at nothing to gain his ends. He was so eager for accurate knowledge that he was entirely ruthless as to how he got them” [27].

**Fig. 5:**
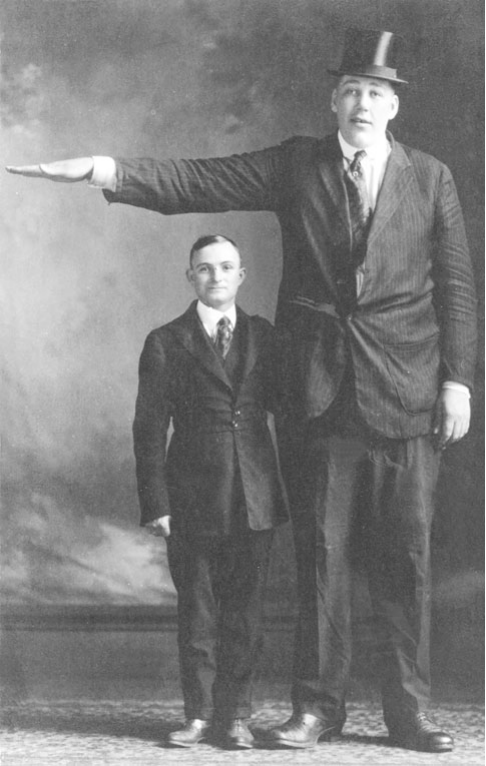
John Aasen (1890–1938) and a normal-sized man. Picture from the collection of Wouter W. de Herder

(5) John (Johan) Aasen (Fig. 5) was born on 5 March 1890 in Minneapolis, Minnesota, USA. He was also known as the “Minneapolis giant”. He was the son of the Norwegian immigrant Kristi Danielsdatter Aasen (Kristi Danielsen). Her height is variously stated as 1.88 m. (6 ft. 2 in.) and 2.20 m. (7 ft. 2½ in.). According to some sources, the father of John Aasen was Nils Jansson Bokke, whose is also variously stated as 1.93 m. (6 ft. 4 in.), 2.29 m. (7 ft. 6 in.) and 2.44 m. (8 ft.) tall. According to other sources, the father of Kristi Danielsdatter Aasen, Daniel Seversen Aasen (“Big Daniel”) was also very tall, reportedly approximately 2.20 m. (7 ft. 2½ in.). However, evidence for this is not available.

By 1917, John Aasen started his circus career. Also known as “Johnny the Gent”, he travelled the world with several circus side shows [28].

Perhaps seeking competition with another American giant of Norwegian descent, Cliff(ord Marshall) Thompson (1904–1955—2.26 m., 7 ft. 5 in.), John Aasen and/or his impresarios rewrote his life story. He changed his birthplace to Numendahl, Norway and said he was educated at the prestigious [but nonexistent] “University of Norway”. John Aasen also stated that family background was the cause of his extraordinary proportions. According to John Aasen, his father was 2.28 m. (7 ft. 6 in.) tall and his mother was 1.91 m. (6 ft. 3 in.) tall. He also made up that his paternal grandfather was the Norwegian giant Henrik Brustad (23 May 1844–5 January 1899), alleged to be 2.52 m. (8 ft. 3 in.) tall but actually 2.26 m (7 ft. 5 in.), who had worked for Phineas Tailor Barnum. According to John Aasen, Henrik Brustad died at the age of 88 years! He thought the prospect of tall people dying young was nonsense, and pointed to Henrik Brustad as proof. (In reality, Brustad died at the age of 55 years) [28].

In 1922, silent film star/director, Hal Roach, needed a tall man for a role in his next silent movie with Harold Lloyd, “Why Worry” (finally released in 1923). This was John Aasen’s breakthrough and—ultimately—his greatest success in the silent movie business. The movie also brought him a new nickname: “Harold Lloyd Giant”. He played a part in several other (silent) movies.

John Aasen was diagnosed with epilepsy in 1931. Despite this diagnosis, he continued drinking large amounts of alcohol at times. A case report of John Aasen at the age of 46 years (1936) was published in 1937 by Dr. Horace Gray [29]. John Aasen never underwent pituitary surgery nor was treated with pituitary radiotherapy. John Aasen died 1 August 1938, only 48 years old, from pneumonia at the Mendocino State Hospital in Mendocino, California, United States. His corpse was placed in a sealed container and sent to Dr. Charles Humberd in Barnard, Missouri, U.S.A. Accordingly, John Aasen had visited Dr. Charles Humberd on several occasions and had willed his body after his death to Dr. Charles Humberd for research purposes and dissection. Dr. Charles Humberd had the skeletons of Johan Aasen and several other giants hanging from the ceiling of his living room up to his death in 1960. John Aasen’s cremated soft tissues were sent to Forest Lawn Cemetery in Glendale, California, U.S.A. ((The grave of Harold Lloyd can also be found here). John Aasen’s skeleton can now be found in Alfred Shryocks Museum in Loma Linda, California, U.S.A. Dr. Kerby C. Oberg of Loma Linda University School of Medicine confirmed and verified, based on the medical case report and radiographs taken by Dr. Horace Gray in 1937, the death certificate and Dr. Charles Humberd’s autopsy-report on John Aasen, that the skeleton in Loma Linda is indeed that of John Aasen.

**Fig. 6:**
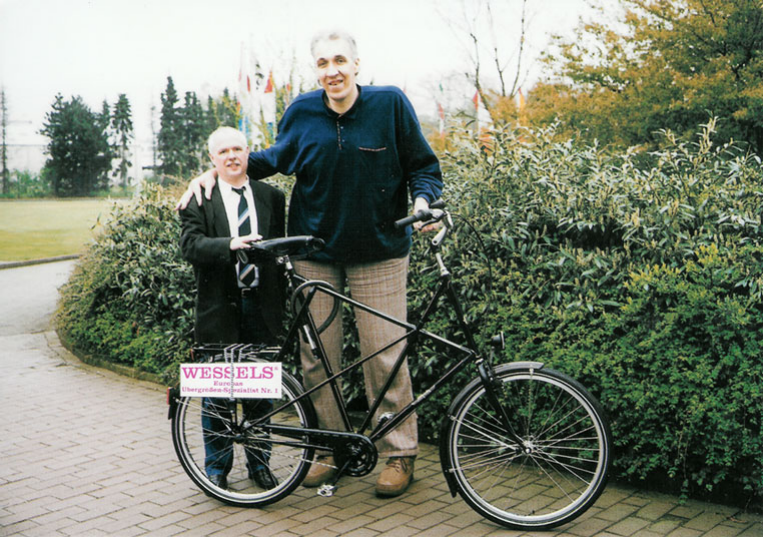
Alexander Sizonenko (1959–2012) and Georg Wessels. Picture kindly provided by Georg Wessels

(6) The acromegalic giant Alexander Alekseyevich Sizonenko (2.41 m., 7 ft 11 in.) (Fig. 6) was born on 20 July 1959 in the village Zaporizhia, Kherson Oblast, Ukraine, and he died recently in St. Petersburg, Ukraine on 5 January 2012. He was a former professional basketball player and played for several local Soviet teams as well as for the national team of the former Soviet Union. In 1991 he was listed in the Guinness book of Records as the tallest man alive. Alexander Sizonenko lived the last years of his life in St. Petersburg, Ukraine. He was divorced and had one son born in 1994.

Although living on a very small monthly pension, he rejected a money offer by the German anatomist Gunther von Hagens to cover his expenses in exchange for publicly displaying his “plastinated” remains after his death.

## Discussion

The life stories of 6, once famous, acromegalic giants who died, respectively, in 1783, 1904, 1911 1912, 1938 and 2012 are presented. The data show that our colleagues who “took care”of, or who were particularly interested in these patients overstepped the (ethical) mark to obtain their corpses. Only one patient voluntarily donated his body to science = a collector and one patient refused to do so despite a very attractive financial offer. Many other skeletons of acromegalic giants have ended up in medical collections of museums all over the world and certainly most did not do so after previous informed consent by the patient during life or their relatives. Several acromegalic giants in the past, while alive, feared the same fate and they, or their relatives, made precautions by trying to guarantee that they would be buried in a very secure fashion (like the giantess Ella Ewing (1872–1913) from La Grange, Missouri, USA and the giant Robert Wadlow (1918–1940) from Alton, Illinois, USA), or cremated. Should endocrinologists therefore continue to warn their acromegalic giants for body snatchers, since still these seem to be amongst us even after more than 2 centuries??!

## References

[1] Doyal L, Muinzer T (2011). Should the skeleton of “the Irish giant” be buried at sea?. BMJ.

[2] Moore W (2012). Byrne’s body should disappear beneath the waves. BMJ.

[3] Shelton D (2012). Don’t forget those who were murdered to order. BMJ.

[4] Smith M, Knusel C, Chamberlain A, Mitchell PD (2012). We cannot change the past, but we can learn from it. BMJ.

[5] Frankcom G, Musgrave JH (1976). The Irish Giant.

[6] McAlister NH (1974). John Hunter and the Irish giant. Can Med Assoc J.

[7] Dobson J (1969). John Hunter.

[8] Cohen B (1993). John Hunter, pathologist. J R Soc Med.

[9] Moore W (2005). The knife man: blood, body snatching, and the birth of modern surgery.

[10] Bergland RM (1965). New information concerning the Irish giant. J Neurosurg.

[11] Wood EJ (1868). Giants and dwarfs.

[12] de Herder WW (2009). Acromegaly and gigantism in the medical literature. Case descriptions in the era before and the early years after the initial publication of Pierre Marie (1886). Pituitary.

[13] Fulton JF (1946). Harvey Cushing.

[14] Cushing H (1912) The pituitary body and its disorders. Clinical States produced by disorders of the hypophysis cerebri. Philadelphia: J. B. Lippincott

[15] Keith A (1911) An inquiry into the nature of the skeletal changes in acromegaly. Lancet 177(4572):993–1002

[16] Cushing H (1930). Neurohypophysial mechanisms from a clinical standpoint. Lancet.

[17] Cushing H (1909). The hypophysis cerebri. Clinical aspects of hyperpituitarism and of hypopituitarism. J Am Med Assoc.

[18] Landolt AM, Zachmann M (1980). The Irish giant: new observations concerning the nature of his ailment. Lancet.

[19] Chahal HS, Stals K, Unterlander M, Balding DJ, Thomas MG, Kumar AV, Besser GM, Atkinson AB, Morrison PJ, Howlett TA, Levy MJ, Orme SM, Akker SA, Abel RL, Grossman AB, Burger J, Ellard S, Korbonits M (2011). AIP mutation in pituitary adenomas in the 18th century and today. N Engl J Med.

[20] Blais JM (1967). Edouard Beaupré, 1881–1904. Can Med Assoc J 1967.

[21] Blais JM (1973) Un géant Canadien célèbre : Édouard Beaupré (1881–1904). Neurochirurgie 19(2):Suppl-2:23–34 4584661

[22] Bertin J (2004). Strange events and more.

[23] Perié A (1995) Le Géant de Mounes. Cercle Genealogique de l’Aveyron

[24] Humberd CD (1937). Giantism. Report of a case. J Am Med Assoc.

[25] Humberd CD (1936). A twenty-five-year-old error in measuring a giant. J Am Med Assoc.

[26] Pendleton C, Wand G, Quinones-Hinojosa A (2010). The autopsy was conducted “Under most inauspicious circumstances:” John Turner, Harvey Cushing’s case XXXII, and his unwitting contributions to the early understanding of acromegaly. Pituitary.

[27] Bliss M (2005). Harvey Cushing: A Life in Surgery.

[28] Hartzman M (2005). American Sideshow.

[29] Gray H (1937). The Minneapolis giant. Ann Intern Med.

